# Is a woman’s first pregnancy outcome related to her years of schooling? An assessment of women’s adolescent pregnancy outcomes and subsequent educational attainment in Ghana

**DOI:** 10.1186/s12978-017-0378-2

**Published:** 2017-10-03

**Authors:** Adriana A. E. Biney, Philomena Nyarko

**Affiliations:** 1 Regional Institute for Population Studies (RIPS), University of Ghana, P. O. Box LG 96, Legon, Accra, Ghana; 2 Ghana Statistical Service (GSS), P. O. Box GP 1098, Head Office Building, Finance Close, Accra, Ghana

**Keywords:** Ghana, Pregnancy outcome, Induced abortion, Female education, Adolescent

## Abstract

**Background:**

Adolescent pregnancy and childbearing pose challenges to young women’s educational attainment. Studies show that while adolescent childbearing reduces educational attainment, not becoming pregnant and resorting to induced abortion when pregnant increases women’s educational levels. This study examined relationships between adolescents’ resolution of their first pregnancies and subsequent educational outcomes, for all women ages 20–49 years and across three age cohorts: 20–29, 30–39 and 40–49 year olds.

**Methods:**

Using the 2007 Ghana Maternal Health Survey (GMHS) dataset, we conducted ANOVA, bivariate and multivariate linear regression analyses on 8186 women ages 20–49 years. Women’s first adolescent pregnancy outcomes were measured as live births, induced abortions, spontaneous abortions or no pregnancy, while educational attainment constituted their years of schooling.

**Results:**

Findings showed years of schooling was highest for women who had induced abortions, and lowest for those who experienced live births. Women with live births as teenagers experienced significantly fewer years of schooling compared to their counterparts who terminated their pregnancies. Also, women with miscarriages and stillbirths exhibited levels similar to those who gave birth. Although women with no teenage births had higher educational levels than their childbearing counterparts, controlling for age at first pregnancy resulted in similar years of schooling compared to those who gave birth. Finally, the 30 to 39 year olds were the only age group whose results contradicted those of all women. These findings may be due to the socio-economic and political events that affected women’s educational attainment at the time.

**Conclusions:**

Childbearing during adolescence does impact women’s educational attainment levels. Therefore, in addition to encouraging young mothers to continue schooling, all other interventions to help keep young girls in school must focus on preventing and/or delaying their adolescent pregnancies.

## Plain English summary

Childbearing during adolescence (between ages 10 and 19) is associated with lower educational attainment while the reverse has been shown for adolescent girls who do not get pregnant or choose to terminate the pregnancy when they do. In this study, the relationship between women’s first adolescent pregnancy outcomes, that is, pregnancies ending in a live birth, induced abortion, spontaneous abortion or no pregnancy, and their current years of schooling was examined. This relationship was also observed across the 20–29, 30–39 and 40–49 year age groups.

The 2007 Ghana Maternal Health Survey dataset was used for the study. The total sample consisted of 8186 women between ages 20 and 49.

Results showed that just over one-half of the women had no pregnancy below age 20; while about 40% of those who were pregnant as teenagers gave birth. Women’s years of schooling also ranged from 0 to 18. Those who had pregnancy outcomes as induced abortions had the most years of schooling and those who had live births had the fewest. These teenage mothers exhibited educational levels similar to those who had spontaneous abortions (miscarriages and stillbirths). When the age at which women had their first pregnancy was included in the analysis, then those who had no pregnancies had similar educational levels as those who had given birth. Also, women between ages 30 and 39 had results that contradicted those of all women.

Interventions to help keep young girls in school must emphasize their delaying pregnancy and childbearing for as long as possible.

## Background

The cultural attitudes and practices pertaining to fertility among most sub-Saharan Africans, and Ghanaians in particular, depict a pronatalist stance [1–3]. This is made even more evident as despite the fact that fertility trends in Ghana over the past three decades indicate a substantial transition from 6.4 to 4.2 births, there has been a stall since 2003 [4] at levels nearly double the replacement value. There are variations in fertility rates across the country and this may be attributable to socio-economic differentials more than geographic ones [2]. One socio-economic indicator that has had a complex effect on fertility in Ghana is formal education [5]. A longstanding relationship exists between the educational attainment of women and their fertility, where generally, higher educational attainment is associated with lower fertility [5–10].

From the literature we understand that women with low educational levels tend to report higher parities, since they start bearing children earlier [6, 7, 11, 12]. In most sub-Saharan countries,^1^ including Ghana, when teenage pregnancies arise, young girls tend to discontinue their education in order to give birth. However, the relationship between pregnancy and schooling also exists in the opposite direction; thus, young girls may be dropping out of school prior to the pregnancy [13]. Reasons for the incidence of school dropouts include their poor academic performance, a lack of funds to continue schooling and family problems, to name a few [4]. There is a negative association between educational aspirations and academic performance, and teenage pregnancy. Those who perform poorly in school and have no aspirations are more likely to experience a teenage pregnancy [14]. They may either drop out before or after the pregnancy. All these established relationships ultimately help us to understand the bi-directional nature of the phenomenon. Findings from the most recent Ghana Demographic and Health Survey (GDHS) show the majority of women (ages 15 to 24) cited financial constraints as their reason for not completing school (38.7%). Only 1.5% of the women mentioned a pregnancy as their reason for dropping out, while 9% stopped school for family reasons or to get married [4]. A qualitative study conducted with young girls in Accra share similar sentiments [15], and this suggests that young women may currently not be dropping out of school due to pregnancy as before.

Much has been written about sub-Saharan African women’s fertility and educational attainment, and studies have gone further to examine other pregnancy outcomes in relation to women’s educational attainment as well [3, 5–9, 12, 16]. Research has shown that women with higher educational levels are likely to have terminated a pregnancy at some point in time as opposed to them giving birth [17]. Bleek [18, 19] suggests that mass education contributed to the increase in induced abortions among the Akan in Ghana, where young women were terminating pregnancies in order to continue with their education [18–20]. Among the reasons for inducing abortions, women often cite continuing with their education as one of them. The 2007 Ghana Maternal Health Survey (GMHS) [21] found that out of the 563 women who had terminated pregnancies between 2002 and 2007, about 10% cited the reason for it as wanting to continue schooling. Women who also want to continue schooling or have educational aspirations are more likely to delay the onset of sexual debut [14], or use contraception effectively to prevent unintended pregnancies [16], and if they happen to get pregnant are more likely to terminate it [22]. Not much research has focused on young girls’ experiences with spontaneous abortions. However, the literature does note that due to their physiological make up as well as a lack of sexual and reproductive health knowledge and access to services when pregnant, adolescent girls are more likely to experience miscarriages and stillbirths than older women [23, 24].

The relationships between these different pregnancy outcomes among adolescents and their educational attainment have rarely been studied together, but doing so provides a means to loosely compare women’s different birth outcomes and their subsequent socio-economic levels. Zabin, Hirsch and Emerson [25] were able to assess longitudinally the relationship between three groups of black adolescent girls who attended a facility to carry out a pregnancy test. One group opted for an abortion; the second opted to give birth while the last group had negative pregnancy test results. Two years later, the study found that young black girls who had opted to carry their pregnancies to term as well as those who were not pregnant suffered negative educational outcomes compared to those who opted for induced abortions [25]. Overall, young girls have different motivations driving them to their pregnancy outcome decisions and these may have repercussions on several aspects of their lives, including their educational attainment [22, 25].

As education continues to become important to women in Ghana, and opportunities for equal access to education, especially at the higher levels, exist, it may have a bearing on their pregnancy decisions and ultimately reproductive health. Ghana’s education policy strives to empower women by promoting gender equality in the classroom. The policies and programs intensified over the past few decades have included making basic education free and compulsory to ensure access to education for all [26, 27]. Affirmative action initiatives have also guaranteed that more women are enrolled in tertiary institutions [28, 29]. Despite these strides, there are still discrepancies between the highest educational attainment levels of men and women, with men exhibiting higher median years of schooling. In addition, literacy rates are higher among 15–24 year old men than their female counterparts (89.3% versus 80.9%) [4] while the tertiary school gender disparity index stands at 0.63^2^ [30]. Female education has been deemed the solution to most problems in society, but unfortunately, there are challenges to some women’s attainment of higher educational levels. Pregnancy outcomes as barriers to adolescent female education are of particular interest to us in this paper.

To the best of our knowledge, not many studies have undertaken a quantitative assessment using nationally representative data from Ghana on the relationship between education and pregnancy outcomes, and whether it is similar across different age cohorts. Similar studies have used either hospital or community-based surveys or qualitative data [11, 25, 31–33]. A nationwide examination of this phenomenon would aid in our understanding of Ghanaian women’s education and pregnancy actions as various policies and programs over 30 years have been implemented to benefit women’s educational attainment. Therefore, in this paper, we aim to examine the first pregnancy outcomes of women in Ghana during their teen years to gain insights into how the outcomes relate to their current educational attainment levels. We also assess this looking at three synthetic cohorts of women, 20–29 year olds, 30–39 year olds and 40–49 year olds, separately, to examine differences in attainment levels by pregnancy outcomes across the three time periods. This paper seeks to show the contribution of all the different outcomes of adolescent pregnancy to female education in Ghana.

### Hypotheses

The study seeks to test four hypotheses based on an understanding of some concepts. First, girls undergo induced abortions to continue their schooling. The issue of selection may exist since it is a certain type of woman that pursues her education and has educational aspirations, and that same ambition may motivate her to undergo an abortion [6, 18, 22]. In addition, external pressure from parents, caregivers, relatives, partners or any persons with influence over their lives, may also motivate the abortion to ensure she does not drop out of school. Based on this we propose this first hypothesis:

1. Women whose first adolescent pregnancies ended in an induced abortion will have higher levels of schooling than those whose first pregnancies ended in a live birth.

Second, fertility and education are very much related as childbearing during adolescence, a time when basic and secondary education should be attained, influences schooling [6, 18]. Specifically, childbearing during adolescence will lead to lower levels of schooling due to one dropping out of school or vice versa [17]. Therefore, not getting pregnant during adolescence should translate into an opportunity for the young girl to attain higher educational levels compared to those who give birth. Therefore, we propose this second hypothesis:

2. Women who did not get pregnant and thus did not give birth during adolescence will have higher levels of schooling than those whose first pregnancies ended in a live birth.

Third, spontaneous abortions provide an avenue through which young girls will have the opportunity to stay in school after losing the pregnancy or fetus. Therefore, we propose this third hypothesis:

3. Women whose first pregnancies ended in a spontaneous abortion or stillbirth will have higher levels of schooling than those whose first pregnancies ended in a live birth.

Finally, educational aspirations and motivations would be greater among the current generation; younger women would seek higher levels due to the many opportunities available to them. The younger women in this sample would have been beneficiaries in some way from the initiatives carried out from the 1990s until the promulgation of the MDGs. Although older women may have been exposed to more years of school than their younger counterparts, they would not have been given access to those opportunities. They may have also faced more pressure to settle down and have children before going back to school compared to the younger cohorts. Based on these concepts we derived the fourth and final hypothesis:

4. Women aged 20 to 29 years would have attained higher levels of schooling than their 30 to 39 and 40 to 49 year old counterparts.

## Methods

### Data and study sample

We used the 2007 GMHS dataset to assess the relationships between young Ghanaian women’s pregnancy outcomes and their educational attainment. To the best of our knowledge the GMHS is the only survey that enables us to access nationally representative information on women’s pregnancy histories as it provides detailed accounts of their pregnancy outcomes. Despite being a dated source, its findings have implications that are still relevant for women’s health at this time. Ethical clearance for the survey was provided by the ICF Macro Institutional Review Board, Maryland, USA.

The surveyed women were sampled using a multi-stage cluster sampling approach. A total of 420 enumeration areas (EAs) were randomly selected across the nation and households in these EAs were then systematically selected. Eligible women, that is, those in the reproductive ages of 15 to 49, were selected from the households to partake in the study. The participation rate was quite high as 98.7% of sampled women were interviewed for the study.

In total, 10,370 women were interviewed; however, this study focuses on two groups of women. The first group consists of all women between ages 20 and 49 years. After adjusting for missing cases for some of the variables, the final sample of women in this group was 8186. Less than 2 % of the sample was removed at this stage. The second group consists of 7208 women ages 20 to 49 years who had ever been pregnant.

### Variables

#### Independent variable

The outcome of women’s first pregnancies as adolescents (pregnancies below age 20) was the predictor variable. Women were asked about the outcomes of each of their pregnancies, whether they ended in a live birth, stillbirth, miscarriage or abortion. We selected women’s first pregnancies that occurred when they were below age 20 (and for some these were their only pregnancies) and re-categorized the outcomes. We combined the responses of women who had miscarriages and stillbirths, and also included women who had not been pregnant as adolescents. Combining miscarriages and stillbirths was necessary, primarily for statistical reasons, which are further discussed as part of the data limitations of the study. Thus, the variable consisted of the following categories: live birth, spontaneous abortion (miscarriage/stillbirth), induced abortion, and no pregnancy. Once again, these refer to outcomes for women whose initial pregnancies occurred when they were teenagers.

#### Dependent variable

A woman’s current educational attainment was measured using ‘number of years of schooling’ at the time of the survey. This was computed using three variables in the dataset, ‘ever been to school’, ‘highest educational level’ and ‘highest grade attained at that level’. Using cross tabulation results from the education variables, we were able to generate the years of schooling variable for women.

#### Covariates

One key variable in the study was age group. Women were divided into three age cohorts: 20–29 years, 30–39 years, and 40–49 years. Socio-cultural variables included religion and ethnicity. Religion was categorized as follows: Catholics, Protestants, Pentecostals/Charismatics, Other Christians, Muslims and ‘Others’. Ethnicity included the four major groups of Akan, Ga-Dangme, Ewe, Mole-Dagbani and also ‘Others’. Socio-economic proxy measures were ‘place of residence’, measured as urban and rural settings, and ‘household wealth’ which was split into five quintiles ranging from poorest to richest. The number of pregnancies a woman has had and her age at first pregnancy were continuous measures, while the number of abortions a woman she has had was re-coded into 4 categories: 0, 1, 2, and 3+. Age at first pregnancy was measured as a continuous variable comprising of the ages at which all women that had ever been pregnant had their first pregnancies.

### Analysis

Bivariate and multivariate linear regression models were conducted to assess relationships between women’s first adolescent pregnancy outcomes and their years of schooling. The mean years of schooling of women across the four pregnancy outcome categories and by age group was also computed and graphed. The linear regression models were run with pregnancy outcome and years of schooling to assess the bivariate relationships between the main independent and dependent variables. Models were run for all women and women who had ever been pregnant, as well as across the three age cohorts. Additionally, multiple linear regression models were conducted to examine the association between pregnancy outcome and years of schooling while controlling for all covariates, with an exception. Only the models conducted with ever been pregnant women included the age at first pregnancy variable. The statistical software package, STATA version 12, was used for the analyses.

### Data limitations

There were a few data limitations to this study. First, the information available on women’s educational attainment levels was inadequate as no question was asked on their schooling at the time of the pregnancy. Thus, there was no evidence to indicate whether pregnancies occurred before respondents ended their education or vice versa; in addition, there was no information to indicate whether the respondent was in school at the time of the survey. The absence of such information does not enable us to make definitive conclusions about the women’s education since some may not have ended their education at the time of the survey. However, we specifically chose women starting from age 20 where we presume they should have finished their secondary education by age 18. Cross tabulation results show that the 20 to 29 year old women have more education than their older counterparts and it is highly likely that some of the younger ones may still be in school.

Second, although stillbirths and miscarriages represent different durations at which women experience pregnancy loss, for example, a fetus may die at delivery and in this case the stillbirth will be almost akin to a live birth, and a miscarriage may take place as early as in the first few weeks of conception, the frequencies of women who had miscarriages (*n* = 181) and stillbirths (*n* = 60) were far too small to leave them as separate categories. Despite this limitation, we argue that ideally both forms of pregnancy loss were unexpected spontaneous and involuntary occurrences, and although duration and timing of the pregnancy loss varied, women were expecting to keep those pregnancies that were ultimately terminated.

Third, it would have been ideal to factor in the timing of the induced and spontaneous abortions which could provide an opportunity to observe its effect on their educational attainment. However, there are many complexities associated with including this variable and this was also beyond the scope of this paper.

Finally, the under-reporting of induced abortions or misreporting them as spontaneous abortions is always a major concern in abortion studies as social desirability and fear of stigma may result in women denying their abortions or reducing the number of repeated abortions they report [34, 35]. In the same manner, there may be under-reporting of adolescent pregnancies, where women refuse to acknowledge their teenage pregnancies due to the shame and stigma that is generally associated with premarital and adolescent pregnancy [33]. Also, women may have reported their pregnancies but misreported the ages at which they had them and this could pose another limitation to us concluding on a true effect of an adolescent pregnancy on women’s educational attainment levels.

## Results

The background characteristics of all women and across age groups in our sample are depicted in Table 1, while those of ever been pregnant women are shown in Table 2. Generally, among all women aged 20 to 49, just over one-half had not been pregnant as teenagers. The next highest proportion of women (39.8%) had live births as teenagers, while 2.9% and 6.5% had spontaneous and induced abortions, respectively. These results are significantly different across the three age cohorts, since as age groups increased the number of women with live births and induced abortions increased, while the proportions that had no teenage births reduced.

**Table 1:** Demographic, socio-economic and socio-cultural characteristics of all women

	All ages		20–29 years		30–39 years		40–49 years	
All Women	N	%	N	%	N	%	N	%
Adolescent First Pregnancy Outcomes								
Live birth	3255	39.8	1124	33.2	1220	42.5	910	47.2
Spontaneous abortion	241	2.9	98	2.9	77	2.7	66	3.4
Induced abortion	532	6.5	232	6.9	189	6.6	111	5.8
No pregnancy	4159	50.8	1932	57.0	1387	48.3	840	43.6
Ethnicity								
Akan	3969	48.5	1627	48.0	1404	48.9	938	48.7
Ga/Dangme	615	7.5	269	7.9	195	6.8	151	7.9
Ewe	1107	13.5	447	13.2	374	13.0	287	14.9
Mole-Dagbani	828	10.1	317	9.4	320	11.2	190	9.9
Other	1667	20.4	727	21.5	580	20.2	360	18.7
Religion								
Catholic	1149	14.0	502	14.8	346	12.0	301	15.6
Protestant	1437	17.6	582	17.2	491	17.1	364	18.9
Pentecostal/Charismatic	2326	28.4	1016	30.0	829	28.8	481	25.0
Muslim	1349	16.5	577	17.0	485	16.9	287	14.9
Other	1926	23.5	710	21.0	722	25.2	493	25.6
Place of residence								
Rural	4680	57.2	1815	53.6	1682	58.5	1183	61.4
Urban	3506	42.8	1571	46.4	1191	41.5	744	38.6
Wealth quintile								
Poorest	1705	20.8	589	17.4	649	22.6	467	24.3
Poor	1750	21.4	709	20.9	608	21.2	433	22.5
Middle	1726	21.1	706	20.8	603	21.0	417	21.7
Rich	1550	18.9	721	21.3	513	17.8	316	16.4
Richest	1455	17.8	662	19.6	500	17.4	293	15.2
Frequency of abortions ever experienced								
0	6766	82.6	2893	85.4	2335	81.3	1537	79.8
1	928	11.3	354	10.5	329	11.5	224	12.7
2	373	4.6	117	3.5	156	5.4	100	5.2
3+	120	1.5	22	0.7	52	1.8	45	2.4
Age Group								
20–29	3387	41.4						
30–39	2873	35.1						
40–49	1927	23.5						
Total	8186	100.0	3387	100.0	2873	100.0	1927	100.0

Close to half of the respondents were Akan, 13.5% were Ewe, about 10% were Mole-Dagbani. Other smaller ethnic groups came together to make up 20% of the sample. These proportions were consistent across the age groups. Women ascribed mostly to the Pentecostal/Charismatic Christian denominations. The proportion of Catholics and Protestants were 14% and 17.6% respectively. The ‘Other’ religion category consisted of Other Christians, Traditionalists/Spiritualists as well as Eastern and other religions.

The majority of respondents resided in rural settings across Ghana, and more 40–49 years olds lived in rural areas than did the 20–29 year olds. The general trend of the wealth quintile showed higher proportions of women in the poorer categories and lower proportions in the richer categories. However, the youngest cohort of women was an exception as they had the smallest proportion in the poorest category and higher proportions compared to the others in the rich and richest wealth categories. Women’s frequency of abortions was generally low. The majority had never had an abortion at the time of the survey. As the number of abortions increased, the proportion of women with repeat abortions reduced drastically. Also, as the age cohorts of women increased, the frequency of abortions increased. This is likely since older women would be exposed to more pregnancies and more opportunities to terminate them. The characteristics of the ever been pregnant only women were very similar to all women, as their proportions across the various categories of the background characteristics were generally comparable. However, removing women who had never been pregnant took out almost half of the younger age cohort residing in urban settings. These were also mostly women in the richer and richest wealth quintiles among the ever been pregnant group of women (see Table 2).

**Table 2:** Demographic, socio-economic and socio-cultural characteristics of women who have ever been pregnant

	All ages		20–29 years		30–39 years		40–49 years	
Ever been pregnant women	N	%	N	%	N	%	N	%
Adolescent First Pregnancy Outcomes								
Live birth	3255	45.2	1124	44.8	1220	43.6	910	47.9
Spontaneous abortion	241	3.3	98	3.9	77	2.7	66	3.5
Induced abortion	532	7.4	232	9.3	189	6.8	111	5.8
No pregnancy	3180	44.1	1052	42.0	1313	46.9	816	42.9
Ethnicity								
Akan	3492	48.4	1206	48.1	1361	48.6	925	48.6
Ga/Dangme	525	7.3	189	7.5	188	6.7	148	7.8
Ewe	960	13.3	314	12.5	363	13.0	283	14.9
Mole-Dagbani	740	10.3	230	9.2	320	11.4	190	10.0
Other	1492	20.7	567	22.6	568	20.3	357	18.8
Religion								
Catholic	997	13.8	364	14.5	336	12.0	297	15.6
Protestant	1249	17.3	412	16.4	477	17.0	360	18.9
Pentecostal/Charismatic	2000	27.7	725	28.9	803	28.7	472	24.8
Muslim	1174	16.3	415	16.6	475	17.0	284	14.9
Other	1789	24.8	591	23.6	708	25.3	489	25.7
Place of residence								
Rural	4402	61.1	1568	62.6	1660	59.3	1174	61.7
Urban	2806	38.9	939	37.5	1139	40.7	729	38.3
Wealth quintile								
Poorest	1641	22.8	531	21.2	644	23.0	466	24.5
Poor	1640	22.8	608	24.3	601	21.5	431	22.7
Middle	1543	21.4	548	21.9	587	21.0	408	21.4
Rich	1301	18.0	487	19.4	500	17.9	313	16.5
Richest	1083	15.0	332	13.3	467	16.7	284	14.9
Frequency of abortions ever experienced								
0	5787	80.3	2013	80.3	2262	80.8	1513	79.5
1	928	12.9	354	14.1	329	11.8	244	12.8
2	373	5.2	117	4.7	156	5.6	100	5.3
3+	120	1.7	22	0.9	52	1.9	45	2.4
Age Group								
20–29	2506	34.8						
30–39	2799	38.8						
40–49	1903	26.4						
Total	7208	100.0	2506	100.0	2799	100.0	1903	100.0

Table 3 displays the mean years of schooling, number of pregnancies, and ages at first pregnancies. As expected, years of schooling were inversely related to age, while number of pregnancies was directly related to age. Women who had ever been pregnant also exhibited less years of schooling and more pregnancies than all women. Finally, the mean age at first pregnancy was high at 19.9 years, and this was roughly the same across the three age cohorts.

**Table 3:** Demographic and socio-economic characteristics of all and ever been pregnant women

	All women				Ever been pregnant women			
Characteristics	Mean	Std Dev	Range	N	Mean	Std Dev	Range	N
All ages								
Years of schooling	5.68	4.54	0–18	8186	5.17	4.34	0–18	7208
Total number of pregnancies	3.71	2.69	0–16	8186	4.22	2.47	1–16	7208
Age at first pregnancy					19.9	3.7	10.1–44.3	7208
20–29 years								
Years of schooling	6.80	4.41	0–18	3387	5.85	4.12	0–18	2506
Total number of pregnancies	1.78	1.58	0–11	3387	2.41	1.36	1–11	2506
Age at first pregnancy					19.5	3.0	10.4–28.9	2506
30–39 years								
Years of schooling	5.00	4.38	0–17	2873	4.91	4.34	0–17	2799
Total number of pregnancies	4.32	2.08	0–14	2873	4.44	1.98	1–14	2799
Age at first pregnancy					20.3	4.1	10.1–37.8	2799
40–49 years								
Years of schooling	4.72	4.57	0–18	1927	4.67	4.54	0–18	1902
Total number of pregnancies	6.20	2.59	0–16	1927	6.28	2.50	1–16	1902
Age at first pregnancy					19.9	3.9	10.7–44.3	1902

**Fig. 1:**
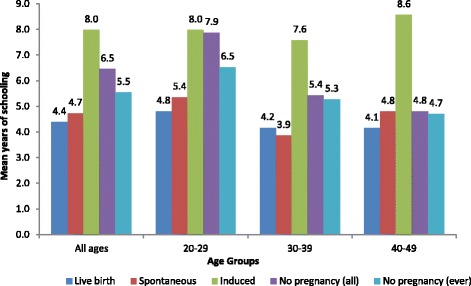
Mean years of schooling for women with the different pregnancy outcomes by age group

Results from the bivariate regression analyses, as indicated in Table 4, show that among all women (20–49 years) and across the three age groups, those with induced abortions and no pregnancies had significantly more years of schooling than women who had live births as teenagers. The relationship was not significant for those who had spontaneous abortions. The same findings are noticed among only the women who had ever been pregnant, except that the beta coefficient value decreases, suggesting less years of schooling once women who have never been pregnant are excluded from the model. The same patterns are shown in Fig. 1, where the mean years of schooling are assessed by the pregnancy outcomes.

**Table 4:** Bivariate analyses showing years of schooling by pregnancy outcome (all and ever pregnant women)

Characteristics	Coef.	Std. Err.	Coef.	Std. Err.	Coef.	Std. Err.	Coef.	Std. Err.
All women	All ages		20–29 years		30–39 years		40–49 years	
Pregnancy Outcomes								
Live Birth (RC)	0.000		0.000		0.000		0.000	
Spontaneous	0.344	0.293	0.545	0.437	−0.304	0.505	0.648	0.570
Induced	3.595***	0.205	3.163***	0.300	3.416***	0.335	4.518***	0.449
No pregnancy	2.070***	0.103	3.074***	0.156	1.282***	0.168	0.670**	0.213
Constant	4.384***	0.077	4.813***	0.124	4.165***	0.123	4.147***	0.148
Adjusted R^2^	0.0649		0.1096		0.0443		0.0491	
N	8186		3386		2872		1927	
Ever been pregnant women	All ages		20–29 years		30–39 years		40–49 years	
Pregnancy Outcomes								
Live Birth (RC)	0.000		0.000		0.000		0.000	
Spontaneous	0.344	0.283	0.545	0.418	−0.304	0.500	0.648	0.565
Induced	3.595***	0.198	3.163***	0.287	3.416***	0.332	4.518***	0.445
No pregnancy	1.158***	0.106	1.716***	0.171	1.106***	0.169	0.558**	0.213
Constant	4.384***	0.074	4.813***	0.119	4.165***	0.122	4.147***	0.147
Adjusted R^2^	0.0489		0.0642		0.0423		0.0505	
N	7208		2506		2799		1902	

*RC* Reference Category, *Coef*. Beta coefficient, *Std. Err*. Standard Error; All estimates are weighted

Note: ***p < 0.001

**p < 0.01

*p < 0.05

+*p* < 0.10

Similar levels are shown between adolescents with live birth and spontaneous abortion experiences. Those with induced abortions have the highest levels of education across the age groups. In addition, once women who had never been pregnant were excluded, the educational levels of women with no pregnancies as teenagers declined.

At the multivariate level of analysis, the same results seen in Table 4 still hold for all women ages 20 to 49 years, where those with induced abortions and no pregnancies have significantly more years of schooling than those with live births (see Table 5). However, upon examination of the age groups separately, we see that it is only among the 30–39 year olds that the relationship with induced abortion and educational attainment does not hold. Among this group who experienced induced abortion, they had similar years of schooling to those who had live births. Results for the covariates were consistent across the age groups. Akans had higher educational levels than the ‘Other’ ethnic groups; all other religious denominations except Protestants had less educational attainment levels compared to the Catholics. Urban residents were also more educated than the rural folks. As wealth quintile increased, the years of schooling of women also significantly increased. The number of pregnancies a woman had was inversely associated with her years of education attained, while the frequency of abortions a woman had was directly associated with her years of schooling. Finally, as women’s ages increased, they had significantly less years of schooling.

**Table 5:** Multivariate analyses showing years of schooling by pregnancy outcome and covariates (all women)

Characteristics	Coef.	Std. Err.	Coef.	Std. Err.	Coef.	Std. Err.	Coef.	Std. Err.
All women	All ages		20–29 years		30–39 years		40–49 years	
Pregnancy Outcomes								
Live Birth (RC)	0.000		0.000		0.000		0.000	
Spontaneous	0.056	0.226	0.337	0.348	−0.319	0.393	0.213	0.442
Induced	0.570**	0.193	0.672*	0.313	0.521	0.317	0.783*	0.399
No Pregnancy	0.697***	0.087	0.972***	0.151	0.336*	0.144	0.362*	0.171
Ethnicity								
Akan (RC)	0.000		0.000		0.000		0.000	
Ga/Dangme	−0.957***	0.147	−0.708**	0.219	−1.182***	0.255	−0.990**	0.303
Ewe	−0.580***	0.116	−0.172	0.178	−0.728***	0.197	−0.865***	0.234
Mole-Dagbani	−2.878***	0.162	−3.144***	0.247	−2.814***	0.266	−2.497***	0.349
Other	−2.662***	0.122	−2.642***	0.184	−2.753***	0.205	−2.510***	0.258
Religion								
Catholic (RC)	0.000		0.000		0.000		0.000	
Protestant	0.038	0.137	0.088	0.208	−0.094	0.240	0.191	0.274
Pentecostal/ Charismatic	−0.601***	0.126	−0.495**	0.188	−0.507*	0.222	−0.815**	0.261
Muslim	−1.515***	0.150	−0.996***	0.225	−1.540***	0.258	−2.482***	0.322
Other	−1.174***	0.128	−1.159***	0.197	−0.990***	0.221	−1.301***	0.255
Place of Residence								
Rural (RC)	0.000		0.000		0.000		0.000	
Urban	0.590***	0.098	0.608***	0.148	0.618***	0.166	0.279	0.211
Wealth Quintile								
Poorest (RC)	0.000		0.000		0.000		0.000	
Poorer	0.901***	0.121	0.886***	0.193	1.154***	0.199	0.619**	0.241
Middle	1.942***	0.128	1.998***	0.204	1.836***	0.211	1.900***	0.255
Richer	2.618***	0.142	2.627***	0.217	2.463***	0.238	2.644***	0.301
Richest	4.327***	0.157	3.787***	0.241	4.637***	0.260	4.695***	0.342
Number of Pregnancies	−0.326***	0.022	−0.616***	0.049	−0.259***	0.035	−0.240***	0.034
Frequency of abortions ever experienced								
0 (RC)	0.000		0.000		0.000		0.000	
1	0.471**	0.137	0.402	0.236	0.513*	0.221	0.779**	0.265
2	1.187***	0.205	1.691***	0.375	0.722*	0.324	1.734***	0.383
3+	1.924***	0.333	2.524**	0.756	1.576**	0.504	1.714**	0.559
Age Group								
20–29 (RC)	0.000							
30–39	−0.704***	0.099						
40–49	−0.328**	0.131						
Constant	6.240***	0.171	6.473***	0.268	5.389***	0.319	5.758***	0.382
Adjusted R2	0.4493		0.4383			0.4255	0.4379	
N	8186		3386		2872		1972	

*RC* Reference Category, *Coef.* Beta coefficient, *Std. Err*. Standard Error; All estimates are weighted

Note: ***p < 0.001

**p < 0.01

*p < 0.05

+p < 0.10

Once the age at first pregnancy was included in the model, which also simultaneously indicated that women with no pregnancies were removed from the analysis, the findings drastically changed (see Table 6). Women with no teenage pregnancy, suggesting their first pregnancy was after age 19, had educational attainment levels similar to those who had live births as teenagers. This indicates that it was the women who had never been pregnant that increased the years of schooling of the group; and thus, having experienced a pregnancy after teenage years gave women similar years of schooling as those who gave birth as adolescents. Once again, those with spontaneous abortions had educational levels similar to those with live births.

**Table 6:** Multivariate analyses showing years of schooling by pregnancy outcome and covariates (ever pregnant women)

Characteristics	Coef.	Std. Err.	Coef.	Std. Err.	Coef.	Std. Err.	Coef.	Std. Err.
All women	All ages		20–29 years		30–39 years		40–49 years	
Pregnancy Outcomes								
Live Birth (RC)	0.000		0.000		0.000		0.000	
Spontaneous	0.049	0.222	0.260	0.337	−0.271	0.390	0.206	0.440
Induced	0.577**	0.189	0.657*	0.304	0.560	0.314	0.792*	0.398
No Pregnancy	−0.102	0.124	0.076	0.216	−0.594**	0.201	0.109	0.242
Ethnicity								
Akan (RC)	0.000		0.000		0.000		0.000	
Ga/Dangme	−1.119***	0.156	−1.057***	0.252	−1.230***	0.258	−0.967**	0.305
Ewe	−0.738***	0.122	−0.466*	0.205	−0.734***	0.198	−0.892***	0.235
Mole-Dagbani	−3.066***	0.167	−3.671***	0.273	−2.885***	0.265	−2.485***	0.349
Other	−2.788***	0.126	−2.975***	0.201	−2.800***	0.205	−2.461***	0.257
Religion								
Catholic (RC)	0.000		0.000		0.000		0.000	
Protestant	0.028	0.144	0.227	0.237	−0.174	0.242	0.119	0.275
Pentecostal/ Charismatic	−0.641***	0.133	−0.425*	0.215	−0.513*	0.223	−0.943***	0.262
Muslim	−1.600***	0.157	−0.973***	0.251	−1.541***	0.258	−2.545***	0.322
Other	−1.190***	0.133	−1.121***	0.218	−0.984***	0.222	−1.393***	0.256
Place of Residence								
Rural (RC)	0.000		0.000		0.000		0.000	
Urban	0.462***	0.103	0.416**	0.164	0.585***	0.166	0.239	0.212
Wealth Quintile								
Poorest (RC)	0.000		0.000		0.000		0.000	
Poorer	0.850***	0.122	0.717***	0.200	1.115***	0.198	0.673**	0.240
Middle	1.809***	0.130	1.629***	0.216	1.862***	0.210	1.913***	0.255
Richer	2.380***	0.146	2.083***	0.234	2.400***	0.239	2.721***	0.301
Richest	4.107***	0.166	3.204***	0.274	4.381***	0.263	4.706***	0.343
Number of Pregnancies	−0.242***	0.023	−0.401***	0.057	−0.175***	0.038	−0.222***	0.036
Frequency of abortions ever experienced								
0 (RC)	0.000		0.000		0.000		0.000	
1	0.675***	0.136	0.601**	0.231	0.611**	0.220	0.832**	0.265
2	1.338***	0.203	1.760***	0.366	0.851**	0.322	1.782***	0.381
3+	1.937***	0.328	2.342**	0.735	1.622**	0.501	1.731**	0.557
Maternal Age	0.119***	0.017	0.180***	0.037	0.169***	0.026	0.044	0.031
Age Group								
20–29 (RC)	0.000							
30–39	−0.593***	0.104						
40–49	−0.363**	0.135						
Constant	3.947***	0.355	3.155***	0.746	2.079***	0.586	4.918***	0.700
Adjusted R2	0.4202		0.3961		0.4237		0.4345	
N	7208		2506		2799		1902	

*RC* Reference Category, *Coef.* Beta coefficient, *Std. Err*. Standard Error; All estimates are weighted

Note: ***p < 0.001

**p < 0.01

*p < 0.05

+p < 0.10

Across the three age groups, we see similar patterns in the relationship between education and pregnancy status, except for the 30 to 39 year olds, where after controlling for the age of mother at the time of her first pregnancy, women with no births as adolescents had significantly fewer years of schooling than those who gave birth. This means that the 30 to 39 year olds not experiencing adolescent pregnancy fared worse than those who had live births. Additionally, as with all women in Table 5, those aged 30 to 39 years with induced abortions as their first adolescent pregnancy outcomes had similar educational attainment levels to those who had given birth.

Age at first pregnancy was an overall significant predictor of educational attainment except among the 40 to 49 year old respondents. This implies that for them, the age of the mother at her first pregnancy did not significantly influence her years of schooling. However, generally, a one year increase in women’s age at first pregnancy resulted in an increase in the number of years of schooling by 0.119 years. The multivariate regression models had high adjusted R^2^ values in the 40s which indicated good fitting models.

## Discussion

The aims of the study were to identify the effect of pregnancy outcome on educational attainment among women in Ghana, and also to assess the relationships between educational attainment and pregnancy outcomes across three age cohorts. At the various levels of analyses, the results show a significant relationship between first adolescent pregnancy outcome and educational attainment. As expected, the findings generally support the first hypothesis but partially support the second hypothesis; therefore, women with induced abortion experiences and no births, respectively, have higher educational attainment levels than those with live births. Without age at first pregnancy being controlled for in the model, women with no teenage pregnancies had significantly higher education; however, including their ages at that time resulted in similar educational attainment values with those who had live births. This implies that it may not be the pregnancy that results in the lower years of schooling but that eventually having a baby, especially at younger ages, may set women back in their education. Pregnancy and childbearing come with concomitant physiological, psychological, social and financial challenges, and without an adequate support system young girls, whether adolescents or young adults, may not be capable of returning back to school after these events take place [36]. Therefore, delaying childbearing well after adolescence should result in girls staying in school longer, especially if their motivations for discontinuing school were due to pregnancy.

The findings, however, fail to support the third hypothesis, since women whose pregnancies ended spontaneously had similar educational levels as those with live births. This could be because the young girls may have planned to keep the pregnancies and expected to give birth but then by chance did not go on to have live births. Thus, their dispositions toward the pregnancy may be similar to those who had live births. Perhaps, they even went on to have subsequent births soon after, as is the case for women who experience miscarriages, stillbirths and neonatal deaths [37–40]. Additionally, the effects of the pregnancy loss, that is, the shame or disappointment, alongside the loss of desire to return back to the classroom, the ails of missing classes and possibly having to repeat a grade, among others, may have resulted in their truncated years of schooling [36].

In their longitudinal study among US female adolescents, Xie et al.’s [14] post hoc analysis showed that better academic performance and higher aspirations resulted in a lower likelihood of teenage pregnancy. This could infer that the brightest and more ambitious pupils, who have something to look forward to, may resort to abortion and continue schooling, while their less studious counterparts stop schooling and ultimately give birth [14]. With this particular dataset, it is difficult to determine whether this phenomenon occurred. In general, schooling can have a protective effect on young people [12] and does this through various means, that is, providing education, skills, monitoring, protection, and so on [41]. Dropping out exposes them to risks of pregnancy and childbearing since if they are no longer enrolled in school they are more likely to get pregnant and keep the pregnancy [14, 41]. On the other hand, schooling also provides networks that may expose young girls to sexual relationships and activity, which may include having sex for school fees and rides to school, as well as some of the luxuries that their friends may have [15, 42, 43]. Generally though, the benefits of formal education far outweigh the risks.

Even after controlling for subsequent pregnancies (if any, which could have resulted in childbirth and induced or spontaneous abortions) after that first pregnancy, the general patterns remain. Thus, we can see that young women in Ghana have accessed abortion services in order to continue with their schooling. This is made evident by the high educational levels of those whose first pregnancies as teenagers were terminated, and even seem to achieve more than those who never got pregnant. The women who gave birth or suffered spontaneous terminations as teenagers seem to be the most disadvantaged groups and this indicates a need to target any conditions that may be putting them at risk of pregnancy. Although not overtly indicated from the findings, the results subtly show that ambition and aspirations of women may be the underlying factor that leads to high educational attainment, confounding the relationship between induced abortion and educational attainment. Aspirations may act as a protective factor for women where ambitious girls are less likely to get pregnant but then if circumstances arise where they get pregnant they are also more likely to abort. Thus, the young girl’s personality or personal agency may drive her to continue her education, thereby resulting in a pregnancy termination or not. Reasons women in Ghana provide for terminating pregnancies vary [44]; and although personal decisions to stay in school or delay/space births are featured, external drivers such as family and partner influence are also very critical to determining how the pregnancy ends [44].

Traditionally, education is seen to contribute to the lowering of fertility through a variety of means, including effective contraceptive uptake [16] where educated women and their partners tend to adopt a modern method of contraception to prevent unintended pregnancies. However, in this study, induced abortion seems to be an alternative for sexually active young women to fall on, providing further evidence that young girls may be resorting to induced abortion as their means of birth control [20]. Additionally, the literature shows no relationship between women’s educational attainment and the safety of the induced abortion methods they employ [45]. Therefore, young girls may be putting themselves at risk as they resort to unsafe induced abortions to terminate their pregnancies in order to stay in school.

Findings across the age groups support hypothesis four, as all women and ever pregnant women, with age groups 30–39 and 40–49, had significantly less years of schooling than the youngest age group. In addition to this, the pregnancy outcome – education relationship was also assessed across three 10-year age cohorts. We see that there were generally similar patterns across the age groups. The only exception was with the 30 to 39 year olds. Among these women, those with induced abortions had similar years of schooling compared to those who had live births. It was rather those with no births that had significantly higher educational attainment level, and this reduced when age at first pregnancy was introduced into the model. In an attempt to understand the age group differences, especially the results among the 30–39 year olds, we briefly reviewed some literature that discussed the economic, social and political contexts that may have possibly influenced female education during the three decades. First, respondents aged 40–49, 30–39 and 20–29 years would have roughly enrolled in primary/basic school in the 60’s-70’s, 70’s-80’s and 80’s-90’s, respectively. During the 90’s, education became a priority, especially for girls, and these reforms may be influencing the high levels younger women are attaining [26, 27]. During the 70’s to the mid 80’s, Ghana went through economic turmoil, a series of coups and eventually a famine, which had a powerful negative effect on women’s lives including education [46, 47]. On the other hand, the 60’s represent more prosperous economic times in Ghana where, post-independence, education was deemed a necessity for development. The government invested in primary, secondary and tertiary education across the country for both boys and girls [47]. Despite this, our results imply that many young women still were not able to attend school during that period.

Our findings also show that educational attainment of women declined as age increased which may be indicative of the changing times and success of programs that have encouraged young girls to stay in school longer. However, in order to meet the Sustainable Development Goals (SDG) that call for access to equitable and quality education for all, a more inclusive system needs to be adopted countrywide to encourage pregnant teenagers to continue schooling, especially after they give birth. There is also the need to strengthen these young girls’ rights to education. In our study, both older and younger women who gave birth faced similar situations when it came to their educational attainment and showed that for decades, dropping out of school has left girls with roughly the same levels of educational attainment.

Current efforts to promote the sexual and reproductive health of young women include establishing adolescent health clubs in secondary schools and adolescent health corners in health facilities, providing access to information, education and communication on adolescent health issues through youth-centered television programs, to name a few [48, 49]. There has been no extensive evaluation of these interventions but generally contraceptive use among adolescents remains low, unmet need is high and adolescent pregnancies have slightly increased from 13.8% in 2008 to 14.2% in 2014 [4, 48–50]. In a context where the median age at sexual debut is 18.4 years and the proportion of 15 to 19 year olds reporting sexual experience increased from 37.3% in 2008 to 42.7% in 2014 [4, 50], adolescent pregnancies are expected. It is these pregnancies, unless aborted, that pose as barriers to young girls’ educational attainment [18, 19, 33]. In-depth studies, especially concerning the sexual and pregnancy-prevention practices and life aspirations (whether educational, career, financial, marital, social, etc.) of adolescent girls, are essential to us understanding the issues holistically in order to provide interventions for their reproductive health needs.

## Conclusions

Overall, among those pregnant at young ages, abortions may result in higher educational attainment, while live births are linked to low levels of education. As expected, women with induced abortion experiences had more education than those who chose to give birth. This finding cuts across for women of all age groups, suggesting that Bleek’s [18] statement may hold for women all over Ghana. Pregnancy at young ages may be setting women back, placing them at a disadvantage in life. Young women with spontaneous abortions had educational levels similar to those with live births and thus must be targeted together for any interventions; especially if those that miscarry eventually go on to have live births. Although women with no adolescent pregnancy had more education, factoring in their pregnancies after adolescence resulted in similar educational levels to those with live births. The issue of why births after adolescence also reduce the years of schooling of women needs to be fully understood through further research. The bi-directional nature of this topic, that is, whether pregnancies occur before girls drop out of school or vice versa, also needs additional investigation, as findings will ultimately generate interventions tailored to address the issues at stake. Finally, as interventions are carried out to help keep young girls in school, stakeholders must also focus on promoting their access to adolescent sexual and reproductive health services to prevent unwanted and mistimed pregnancies.

## Footnotes

1 One exception to this is in South Africa where young women may get pregnant as teenagers but are encouraged to continue schooling after giving birth [51].

2 The index is less than one which indicates that men are more represented in higher education than women.

## References

[1] Caldwell JC, Caldwell P (1987). The Cultural Context of High Fertility in sub-Saharan Africa. Popul Dev Rev.

[2] Agyei-Mensah S, Owoo NS (2015). Explaining regional fertility variations in Ghana. J Popul Res.

[3] Agyei-Mensah S (2006). Fertility Transition in Ghana: Looking Back and Looking Forward. Popul Space Place.

[4] GSS GHS, ICF International (2015). Ghana Demographic and Health Survey 2014.

[5] Benefo KD (2005). Child schooling and contraceptive use in rural Africa: A Ghanaian case study. Popul Res Policy Rev.

[6] Johnson-Hanks J (2003). Education, Ethnicity, and Reproductive Practice in Cameroon. Popul English Ed.

[7] Dodoo FN-A, Horne C, Biney A (2014). Does education mitigate the adverse impact of bridewealth on women’s reproductive autonomy?. Genus.

[8] Bongaarts J (2010). The causes of educational differences in fertility in Sub-Saharan Africa. Vienna Yearbook of Popul Res.

[9] Kravdal Ø (2002). Education and fertility in sub-Saharan Africa: Individual and community effects. Demography.

[10] Colclough C (2012). Education, poverty and development – mapping their interconnections. Comp Educ.

[11] Morhe ESK, Tagbor HK, Ankobea FK, Danso KA. Reproductive experiences of teenagers in the Ejisu-Juabeng district of Ghana. Int J Gynecol Obstet. 2012;118(2):137–40. Available from: doi: 10.1016/j.ijgo.2012.03.035 10.1016/j.ijgo.2012.03.035 22652480

[12] Neal SE, Chandra-Mouli V, Chou D. Adolescent first births in East Africa: disaggregating characteristics, trends and determinants. Reprod Health. 2015;12(1):13. Available from: https://doi.org/10.1186/1742-4755-12-13. 10.1186/1742-4755-12-13 PMC4429933 25971731

[13] Takyi BK, Addai I (2002). Religious Affiliation, Marital Processes and Women’s Educational Attainment in a Developing Society. Sociol Relig.

[14] Xie Y, Harville EW, Madkour AS (2014). Academic performance, educational aspiration and birth outcomes among adolescent mothers: A national longitudinal study. BMC Pregnancy Childbirth.

[15] Henry R, Fayorsey C. Coping with pregnancy: Experiences of adolescents in Ga Mashi, Accra. ORC Macro: Calverton; 2002. Available from: https://dhsprogram.com/pubs/pdf/QRS5/copingwithpregnancy.pdf.

[16] Ainsworth M, Beegle K, Nyamete A (1996). The impact of women’s schooling on fertility and contraceptive use: A study of fourteen sub-Saharan African countries. World Bank Econ Rev World Bank.

[17] Johnson-Hanks J (2002). The lesser shame: Abortion among educated women in southern Cameroon. Soc Sci Med.

[18] Bleek W (1990). Did the Akan resort to abortion in pre-colonial Ghana? Some conjectures. Africa (Lond).

[19] Bleek W (1981). Avoiding shame: the ethical context of abortion in Ghana. Anthropol Q.

[20] Anarfi JK, Basu AM (2003). The role of local herbs in the recent fertility decline in Ghana: contraceptives or abortifacients?. The Sociocultural and Political Aspects of Abortion: Global Perspectives.

[21] GSS GHS, Macro International (2009). Ghana Maternal Health Survey 2007.

[22] Ortiz CG, Vazquez NE (1987). Adolescent pregnancy: effects of family support, education, and religion on the decision to carry or terminate among Puerto Rican teenagers. Adolescence.

[23] Ringheim K, Gribble J. Improving the reproductive health of sub-Saharan Africa’s youth: a route to achieve the millenium development goals. Washington: Population Reference Bureau; 2010. Available from: http://www.prb.org/pdf10/youthchartbook.pdf.

[24] Chantrapanichkul P, Chawanpaiboon S. Adverse pregnancy outcomes in cases involving extremely young maternal age. Int J Gynecol Obstet. 2013;120(2):160–4. Available from: doi: 10.1016/j.ijgo.2012.08.024. 10.1016/j.ijgo.2012.08.024 23182803

[25] Zabin LS, Hirsch MB, Emerson MR (1989). When urban adolescents choose abortion: effects on education, psychological status and subsequent pregnancy. Fam Plan Perspect.

[26] Osei RD, Owusu GA, Asem FE, Afutu-Kotey RL. Effects of capitation grant on education outcomes in Ghana. Accra, Inst Soc Econ Res . 2009;(April). Available from: http://www.researchgate.net/profile/Robert_Osei/publication/228871302_Effects_of_capitation_grant_on_education_outcomes_in_Ghana/links/0c96052287a3b36ec8000000.pdf\nhttp://www.researchgate.net/profile/Robert_Osei/publication/228871302_Effects_of_Capitatio.

[27] Nudzor HP (2012). Exploring the policy implementation paradox: using the Free Compulsory Universal Basic Education (fCUBE) policy in Ghana as an exemplar. Int J Qual Stud Educ.

[28] Morley L, Leach F, Lugg R (2009). Democratising higher education in Ghana and Tanzania: Opportunity structures and social inequalities. Int J Educ Dev.

[29] World Bank (2009). Literature Review on Equity and Access to Tertiary Education in the Africa Region.

[30] Kaneda T, Bietsch K (2015). 2015 World Population Data Sheet.

[31] Adanu RMK, Ntumy MN, Tweneboah E (2005). Profile of women with abortion complications in Ghana. Trop Dr.

[32] Schwandt HM, Creanga AA, Danso KA, Adanu RMK, Agbenyega T, Hindin MJ. A comparison of women with induced abortion, spontaneous abortion and ectopic pregnancy in Ghana. Contraception. 2011;84(1):87–93. Available from: doi: 10.1016/j.contraception.2010.10.011 10.1016/j.contraception.2010.10.011 21664516

[33] Aziato L, Hindin MJ, Maya ET, Manu A, Amuasi SA, Lawerh RM, et al. Adolescents’ responses to an unintended pregnancy in Ghana: A qualitative study. J Pediatr Adolesc Gynecol. 2016;29(6):653–8. Available from: http://www.sciencedirect.com/science/article/pii/S1083318816300869. 10.1016/j.jpag.2016.06.005 27346553

[34] Mote CV, Otupiri E, Hindin MJ (2010). Factors associated with induced abortion among women in Hohoe. Ghana Afr J Reprod Health.

[35] Shah I, Åhman E (2010). Unsafe abortion in 2008: Global and regional levels and trends. Reprod Health Matters.

[36] Ananga ED. Typology of school dropout: The dimensions and dynamics of dropout in Ghana. Int J Educ Dev. 2011;31(4):374–81. Available from: doi: 10.1016/j.ijedudev.2011.01.006.

[37] Davanzo J, Hale L, Razzaque A, Rahman M (2007). Effects of interpregnancy interval and outcome of the preceding pregnancy on pregnancy outcomes in Matlab, Bangladesh. BJOG An Int J Obstet Gynaecol.

[38] Love ER, Bhattacharya S, Smith NC, Bhattacharya S (2010). Effect of interpregnancy interval on outcomes of pregnancy after miscarriage : retrospective analysis of hospital episode statistics in Scotland. Br Med J.

[39] Makhlouf MA, Clifton RG, Roberts JM, Myatt L, Hauth JC, Leveno KJ (2014). Adverse pregnancy outcomes among women with prior spontaneous or induced abortions. Am J Perinatol.

[40] Nyarko PE, Madise N, Diamond I. Child Loss and Fertility Behaviour in Ghana . Southampton; 2003. SSRC Applications and Policy Working Paper Series, No. A03/08. Available from: http://eprints.soton.ac.uk/8143/1/8143-01.pdf.

[41] Rosenberg M, Pettifor A, Miller WC, Thirumurthy H, Emch M, Afolabi SA, Kahn K, Collinson M, Tollman S (2015). Relationship between school dropout and teen pregnancy among rural South African young women. Int J Epidemiol.

[42] Oruko K, Nyothach E, Zielinski-gutierrez E, Mason L, Alexander K, Vulule J (2015). “He is the one who is providing you with everything so whatever he says is what you do”: A qualitative study on factors affecting secondary schoolgirls’ dropout in rural Western Kenya. PLoS One.

[43] Jukes M, Simmons S, Bundy D (2008). Education and vulnerability: the role of schools in protecting young women and girls from HIV in southern Africa. AIDS.

[44] Biney A, Atiglo DY. Examining the association between motivations for induced abortion and method safety among women in Ghana. Women Health. 2016; Available from: http://www.tandfonline.com/doi/abs/10.1080/03630242.2016.1235076. 10.1080/03630242.2016.1235076 27636891

[45] Sundaram A, Juarez F, Bankole A, Singh S (2012). Factors Associated with Abortion-Seeking and Obtaining a Safe Abortion in Ghana. Stud Fam Plan.

[46] Derose LF, Wu L, Dodoo FNA (2010). Inferring gender-power: Women’s schooling and relative spousal influence in childbearing in Ghana. Genus.

[47] Akyeampong K. 50 years of educational progress and challenge in Ghana. Consortium for Research on Educational Access, Transitions and Equity (CREATE). Sussex; 2010. Research Monograph: Pathways to Access Series, No. 33. Available from: http://www.create-rpc.org/pdf_documents/PTA33.pdf

[48] Ghana Health Service. Family Health Division - 2015 Annual Report. Accra, Ghana; 2016. Available from: http://www.ghanahealthservice.org/downloads/2015_FAMILY_HEALTH_DIVISION_ANNUAL_REPORT.pdf

[49] Ghana Health Service. Family Health Division - 2016 Annual Report. Accra, Ghana; 2017. Available from: http://www.ghanahealthservice.org/downloads/FHD_2016_ANNUAL_REPORT_Final_June%2019.2017%20nat%20final.pdf.

[50] Ghana Statistical Service (GSS), Ghana Health Service (GHS), ICF Macro (IM). Ghana Demographic and Health Survey, 2008. Accra, Ghana: GSS, GHS, and ICF Macro; 2009. Available from: https://www.dhsprogram.com/pubs/pdf/FR221/FR221[13Aug2012].pdf.

[51] Manzini N (2001). Sexual initiation and childbearing among adolescent girls in KwaZulu Natal. South Africa Reprod Health Matters.

